# Does Employment Status Matter for Emerging Adult Identity Development and Life Satisfaction? A Two-wave Longitudinal Study

**DOI:** 10.1007/s10964-024-01992-x

**Published:** 2024-05-15

**Authors:** Kai Hatano, Shogo Hihara, Manabu Tsuzuki, Reiko Nakama, Kazumi Sugimura

**Affiliations:** 1 Graduate School of Sustainable System Science, Osaka Metropolitan University, Osaka, Japan; 2 Faculty of Business Administration, Matsuyama University, Matsuyama, Japan; 3 Faculty of Letters, Chuo University, Hachioji, Japan; 4 Graduate School of Education, Hyogo University of Teacher Education, Hyōgo, Japan; 5 Graduate School of Humanities and Social Sciences, Hiroshima University, Higashihiroshima, Japan

**Keywords:** late emerging adulthood, identity, employment status, life satisfaction, Japan

## Abstract

Late emerging adulthood is pivotal for identity exploration and development and is interrelated with life satisfaction. In the development of identity and life satisfaction, it is important to have a stable employment status that supports the foundation of life. However, the interrelationships among identity, life satisfaction, and employment status in late emerging adulthood are unclear. Using a two-wave longitudinal survey, this study examined identity development and whether the relationship between identity and life satisfaction varies by employment status. Participants included 875 Japanese emerging adults (65.5% women, *M*_*age*_ = 24.74, *SD*_*age*_ = 0.44). Multivariate analysis of variance showed that identity synthesis decreased for those who lost their jobs or those who changed from being full-time to part-time employment. Additionally, individuals with full-time employment had higher identity synthesis and lower confusion than those with part-time or no employment. Multi-group path analysis indicated that identity synthesis was positively associated with life satisfaction and confusion was negatively associated, and these relationships did not differ by employment status. These findings suggest that identity development in late emerging adulthood varies by employment status and that the association between identity and life satisfaction may not be moderated by employment status. Overall, this study contributes to elucidating the characteristics of identity development along employment status and its relationship to life satisfaction in late emerging adulthood.

## Introduction

In countries with high enrollment in higher education, emerging adulthood (i.e., 18–29 years) indicates a transition from school to society, during which individuals explore their identities, ask themselves who they are in society, and find answers to questions regarding their identity (Arnett et al., 2014). These identity exploration processes are intercorrelated with subjective well-being. Emerging adults with a clear sense of identity have a definite perspective on and are satisfied with life, whereas those with a confused sense of identity feel subjectively dissatisfied with their lives (Hatano et al., 2022). While ample research has examined the development of identity and its relationship with life satisfaction (De Lise et al., 2023), identity development in late emerging adulthood (i.e., 25–29 years) remains unexplored. Consequently, two gaps remain. First, it is unclear whether identity changes progressively during late emerging adulthood. Investigating it would add new evidence to the previously revealed knowledge of identity development in early to middle emerging adulthood. Second, the effect of employment status on identity development and the relationships between identity and life satisfaction have not been examined. Late emerging adulthood is the time for most emerging adults to obtain a job, and obtaining a full-time job strongly influences emerging adults’ identity development and the relationship between identity and life satisfaction. Examining the interplay among employment status, identity, and life satisfaction indicates that challenges in employment may hinder the healthy identity development of emerging adults. Moreover, this analysis offers an opportunity to approach developmental issues from both psychological and social perspectives (Côté & Levine, 2016). To address these research gaps, this study examined identity development and the association between identity and life satisfaction, focusing on the moderating effect of employment status among Japanese late emerging adults.

### Identity Development in Emerging Adulthood

Identity is the sense of continuity and sameness of the self over time (i.e., identity synthesis), whereas a sense of a fragmented and inconsistent self is considered as identity confusion (Erikson, 1968). An individual’s identity is not always fully synthesized or fully confused. For example, an emerging adult may work in sales to lead a prosperous life. However, they want to do social work, albeit at a lower salary. In this case, they have a clear sense of identity (i.e., synthesis) in that they are financially well off, but a confused sense of identity in not getting the job that they want, leading to high identity synthesis and confusion. Therefore, identity synthesis and confusion are not on a continuum but coexist within the individual (Marcia, 2002).

Identity development refers to a decrease in confusion and an increase in synthesis within an individual (Erikson, 1968). Specifically, identity development occurs during the transition from schooling to the workforce, particularly in countries with elevated rates of higher education enrollment (Arnett et al., 2014). Throughout this period of career exploration, adolescents and emerging adults engage in self-reflection regarding their abilities, contemplate their future aspirations, and make commitments to particular career paths (Super, 1980). In this process, adolescents and emerging adults may experience aspects of confusion or synthesis within their identities (Schwartz et al., 2005). A cross-sectional study of Belgian adolescents and emerging adults aged 12–25 indicated higher synthesis and lower confusion in emerging adults aged 23 years than in adolescents aged 12–16 (Bogaerts et al., 2021). A longitudinal study of Japanese adolescents and emerging adults aged 12–24 years showed high synthesis and confusion from the age of 12 to around 18 years; however, after 18 years, confusion was higher than synthesis (Hatano et al., 2022). These two studies suggest that patterns of identity change vary between countries, and that identity confusion persists around the age of 25 in Japan.

However, these studies are limited to examining identity development from adolescence to middle emerging adulthood and have not examined identity development in late emerging adulthood. To better understand the patterns of identity development in emerging adulthood, research on identity development in late emerging adulthood is needed. Therefore, this study focuses on the identity development in late emerging adulthood.

### The Relationship Between Identity Development and Employment Status in Japanese Late Emerging Adults

Employment status is intricately intertwined with identity formation. Late emerging adulthood marks a phase in which individuals have completed their higher education, placing significant economic importance on work in their daily lives, with work occupying a substantial portion of their time (Super, 1980). For late emerging adults, assurance regarding their job’s alignment with their aspirations and its social stability is pivotal for a fulfilling life (Erikson, 1968). Consequently, employment status—whether full-time, part-time, or unemployment—is closely associated with a sense of social security (Conway & Briner, 2002). Full-time employment not only offers financial stability but also confers social recognition through higher wages and greater social validation (Wilkinson et al., 2003). The sense of security regarding future prospects based on employment status significantly contributes to self-consistency, while employment-related anxieties erode this sense (Côté & Levine, 2016). Therefore, attaining a meaningful job or full-time employment in one’s career trajectory is anticipated to play a pivotal role in the identity development of late emerging adults.

In Japan, full-time employment is essential to the economic and social stability of emerging adults. The average annual income of emerging adults in their 20 s varies by about 1 million yen, from 2.5 to 3.5 million yen for emerging adults with part-time and full-time employment, respectively (Statistics Bureau, Ministry of Internal Affairs and Communications, 2022). Regarding social guarantees, full-time jobs are fixed-term and offer compensation in cases of illness, whereas part-time jobs do not compensate in illness. Furthermore, part-time workers are more worried about their future than are full-time workers (Cabinet Office, 2022). These social guarantees have a strong influence on emerging adults’ sense of identity. Therefore, obtaining a full-time job is important for Japanese emerging adults to have a sense of social stability.

However, the effects of employment status on identity development have not been investigated. Individuals with full-time employment may have higher levels of synthesis and lower levels of confusion than those with part-time or no employment. Furthermore, if identity is clarified by employment status, individuals who obtain a job or have a more stable job (e.g., part-time employment converts to full-time employment) will experience a more pronounced progression of identity development than those who do not. Conversely, individuals who lose their jobs or have less-stable jobs (e.g., full-time employment converts to part-time employment) will have more pronounced identity confusion than those who do not.

### Identity, Life Satisfaction, and Employment Status

Identity synthesis is a state with a clear goal for the future (Luyckx et al., 2008), which leads to the regulation of emotions and cognition and adaptation of life toward the goal (Crocetti et al., 2013). Contrastingly, identity confusion implies unclear goals and inability to self-regulate and live adaptively (Bogaerts et al., 2021). Identity synthesis and confusion are associated with a subjective sense of satisfaction or dissatisfaction with life (e.g., Diener et al., 2005). Identity synthesis is positively associated with life satisfaction, and confusion is negatively associated with it (Schwartz et al., 2015). Furthermore, from early adolescence to middle emerging adulthood, individuals with higher identity synthesis and confusion scores had higher and lower life satisfaction scores, respectively (Hatano et al., 2022), suggesting that identity is strongly related to life satisfaction across developmental stages.

The differences and changes in employment status may also affect the relationship between identity and life satisfaction. For emerging adults, work provides the economic and social foundation for living as an individual (Wilkinson et al., 2003). Even if one has clear goals for the future (i.e., synthesis), they are insufficient to be satisfied with life. For example, even if an individual has a clear goal of wanting to work in welfare, it is difficult for them to feel life satisfaction if they are unable to find such a job or if they are economically and socially insecure, as happens in a part-time job. However, if they have a stable, full-time job in welfare, the clear goal of working in welfare is significantly associated with life satisfaction. Individuals with stable employment status may have a stronger association between identity and life satisfaction than those who do not. Therefore, focusing on the effect of employment status on identity development and its moderating effect on the relationship between identity and life satisfaction may reveal an interrelationship between individual psychological development and social status.

## Current Study

Late emerging adulthood is a time of identity development, and identity is strongly associated with life satisfaction. However, how identity develops in late emerging adulthood, and how employment status impacts identity development and the association between identity and life satisfaction are unclear. With these research objectives, this study examined identity development and the association between identity and life satisfaction based on a two-wave longitudinal survey focusing on the moderating effects of employment status. Regarding identity development, this study examined changes in identity and how these changes are moderated by employment status. It was expected that identity synthesis would increase and confusion would decrease in late emerging adulthood (Hypothesis 1). Furthermore, it was expected that individuals who obtained a job or improved their employment status would have increased synthesis and decreased confusion compared to those who did not change their employment status (Hypothesis 2). Whereas, individuals who have lost their jobs or whose employment status worsened would have decreased synthesis and increased confusion compared to those whose employment status was unchanged (Hypothesis 3). Furthermore, individuals with full-time jobs were expected to have a more developed identity than those with part-time or no jobs (Hypothesis 4). Regarding the association, it was expected that individuals with full-time jobs would have a stronger association between identity and life satisfaction and lower intra-individual correlations than those with part-time or no employment (Hypothesis 5).

## Method

### Participants

Participants were 4636 Japanese emerging adults (61.1% women; *M*_*age*_ = 24.75, *SD*_*age*_ = 0.43). Of them, 26.6% (*n* = 1231) participated in the second survey after four years (71.7% women; *M*_*age*_ = 27.74; *SD*_*age*_ = 0.44). Consistent with the study purpose, those with unknown employment status at the time of each survey and who were housewives or students at the second time point were excluded. Finally, 875 emerging adults (65.5% women, *M*_*age*_ = 24.75, *SD*_*age*_ = 0.44 were included in the analysis. At Time 1, 55.8% had a full-time job, 28.5% had a part-time job, 9.4% were unemployed, and 6.4% were university students. At Time 2, 65.3% had a full-time job, 26.6% had a part-time job, and 8.1% were unemployed. In Japan, after finishing school, 70.5%, 24.0%, 4.5%, and 1.0% of emerging adults worked full-time, worked part-time, were unemployed, or had an unknown status, respectively (Employment, Wage and Labour Welfare Statistics Office, 2018). In comparison to these data for Japan as a whole, participants were slightly more likely to work part-time or be unemployed as emerging adults. Table S1 lists the residential regions, household incomes, and educational backgrounds of participants.

### Procedure

Data were obtained from the International Adolescent Psychology Project in Japan (IAPP-J; http://web.hyogo-u.ac.jp/nakama/iappj/src/index.html), which comprised two waves conducted in 2015 and 2019 at four-year intervals. The data are outsourced to an online research company that secures large monitors and is one of the largest data marketing research companies in Japan. People over 18 years are registered with the company, which collects data according to the client’s preferences. For this study, the company was asked to collect data from the maximum possible number of individuals aged 24–25 years, if the budget permits. When a request for data was received, the company informed the intended registrants about the survey. Informed consent was obtained, and the information on the time taken to complete the survey, its content, and the right to stop responding in the middle of a response was provided. Participants who agreed to these statements received an email with the URL to complete the survey. Participants who completed the survey were paid 40 JP\ (approximately $US0.40) per survey.

### Missing Data

T-tests were conducted to examine the impact of attrition. Comparing T1 synthesis, confusion, and life satisfaction scores between individuals who participated only in T1 survey and those who participated in both surveys, no variable was significant (synthesis: *t*(4401) = 0.585, *p* = 0.558, *d* = 0.02; confusion: *t*(4370) = 1.264, *p* = 206, *d* = 0.04; and life satisfaction, *t*(4448) = −0.325, *p* = 0.745, *d* = 0.01). Further, the data for the 875 emerging adults in the analysis included 4.2–5.9% of missing data. Therefore, Little’s (1988) MCAR test was performed to determine whether the missing data were random. The results indicated that missing data may have occurred at random (*χ*^2^(82) = 80.67, *p* = 0.52). For structural equation modeling, the full information maximum likelihood was used to deal with missing values, and the maximum likely robust estimation method was employed.

### Measures

#### Identity

The Erikson Psychosocial Stage Inventory (Rosenthal et al., 1981; Japanese validation by Hatano et al., 2022) was used. This measure comprises 12 items: six for synthesis (e.g., “I have a clear idea of what I want to be”) and six for confusion (e.g., “I change my opinion of myself a lot”), rated on a five-point Likert-type scale from 1 (*completely untrue*) to 5 (*completely true*). Regarding synthesis, the item “I have a strong sense of what it means to be female/male” was not used because it was considered undesirable from a gender perspective. Cronbach’s alpha values for the two waves were 0.77 and 0.80 for synthesis and 0.73 and 0.78 for confusion, respectively, indicating acceptable internal consistency.

#### Life Satisfaction

The Satisfaction with Life Scale (Diener et al., 1985; Japanese validation by Hatano et al., 2022) was used. This measure comprises five items rated on a five-point Likert-type scale ranging from 1 (*completely untrue*) to 7 (*completely true*). A sample item is “In most ways, my life is close to my ideal.” In T1 and T2, the Cronbach’s alphas were 0.89 and 0.91, respectively, indicating great internal consistency.

#### Employment Status

Individuals with the same or changed employment status were identified at T1 and T2. Participants were divided into five groups based on their employment status at two different times. Those with a full-time job at both times were called the *full-time* group (*n* = 452), those with part-time jobs at both times were called the *part-time* group (*n* = 181), and those who were unemployed at both times were called the *unemployed* group (*n* = 61). Participants who moved from part-time, unemployed, or student status to either part-time or full-time employment between the two times were labeled the *improved employment* group (*n* = 139). *Improved employment* participants were those who changed from being unemployed to part-time (*n* = 13) or full-time (*n* = 8) employed, part-time to full-time (*n* = 64) employed, and students to part-time (*n* = 9) and full-time (*n* = 45) employed. Conversely, participants who shifted from formal to *part-time* or unemployed status between the two times were labeled the *worsened employment status* group (*n* = 40). *Worsened employment* participants included those who changed from being full-time to part-time (*n* = 30) employed, full-time employed to unemployed (*n* = 4), part-time employed to unemployed (*n* = 4), and students to unemployed (*n* = 2).

### Statistical Analysis

Analyses of structural equation models and path analysis were conducted using Mplus 8.4 (Muthén and Muthén 1998–2021) and multivariate analysis of variance (MANOVA) was conducted using SPSS version 25.0 (SPSS Inc., Chicago, IL).

As a preliminary analysis, three types of measurement invariance tests (i.e., configural, metric, and scalar invariance) were performed to confirm that the two-factor model of identity and the single-factor model of life satisfaction were equivalent across measurement waves. To test for measurement invariance, confirmatory factor analyses (CFAs) were performed using a parceling approach for identity. Parceling is recommended when the scale has more than five items for each construct and the sample size is large (Bagozzi & Heatherton, 1994). Using several indicators in CFAs often results in many correlated residuals, which reduces both the fit of the model and the utility of the latent variable in capturing the construct of interest (Marsh et al., 1998). Therefore, the model was constructed to have no more than five items or parcels for the synthesis and confusion latent variables. Specifically, one parcel (i.e., a combination of two items) and three items were loaded on the synthesis factor, and two parcel (i.e., a combination of two items) and two items were loaded on the confusion factor (Figure S1).

Following Dimitrov (2010), the standard procedure to test for measurement invariance was used: (a) configural invariance (the same number of factors and patterns of fixed and freely estimated parameters across time points); (b) metric invariance (equivalence of factor loadings across time points, indicating that respondents from each time point attribute the same meaning to the latent construct); and (c) scalar invariance (equivalence of factor loadings and intercepts across time points, indicating that respondents from each time point attribute the same meaning to the latent construct and intercept).

For optimal model fit, the comparative fit index (CFI) and the root mean square error of approximation (RMSEA) should be greater and less than 0.95 and 0.05, with values greater and less than 0.90 and 0.08, respectively, representing a reasonable fit (Kline, 2015). The Satorra-Bentler χ^2^ difference test (*S-Bχ*^2^; Cheung & Rensvold, 2002) and the differences in CFI (ΔCFI) and RMSEA (ΔRMSEA) between models were used to test whether model fit was equivalent across time. The null hypothesis of invariance was rejected if the differences in the model fit indices exceeded the following criteria: significant changes in S-Bχ^2^ at *p* < 0.05, ΔCFI ≥ 0.010, and ΔRMSEA ≥ 0.015 (Kline, 2015).

To examine whether identity development varied by employment status, a 5 (employment status: full-time, part-time, unemployed, improved employment, and worsened employment) × 2 (T1 and T2) repeated-measures MANOVA was conducted. Then, using only the data from T2, multi-group path analysis was used to examine whether the relationship between identity and life satisfaction was moderated by employment status. Assuming a model in which identity predicts life satisfaction, five groups were set up for employment status (i.e., full-time, part-time, unemployed, improved employment, and worsened employment). To examine differences between the five groups in the associations of synthesis and confusion with life satisfaction, Wald chi-square tests were conducted.

## Results

### Measurement Invariance Test

For measurement invariance test, a two- and one-factor model were fitted for identity and life satisfaction, respectively. The results of the longitudinal invariance tests for identity clearly indicated that all hierarchical levels of invariance (configural, metric, and scalar) could be established (Table S1). For life satisfaction, the ΔCFI and ΔRMSEA values met the criteria; however, the S-Bχ^2^ test results were significant (Table S2). Therefore, the hierarchical levels of invariance (configural, metric, and scalar) could be established based on these criteria.

### Identity Development in Employment Status

**Fig. 1:**
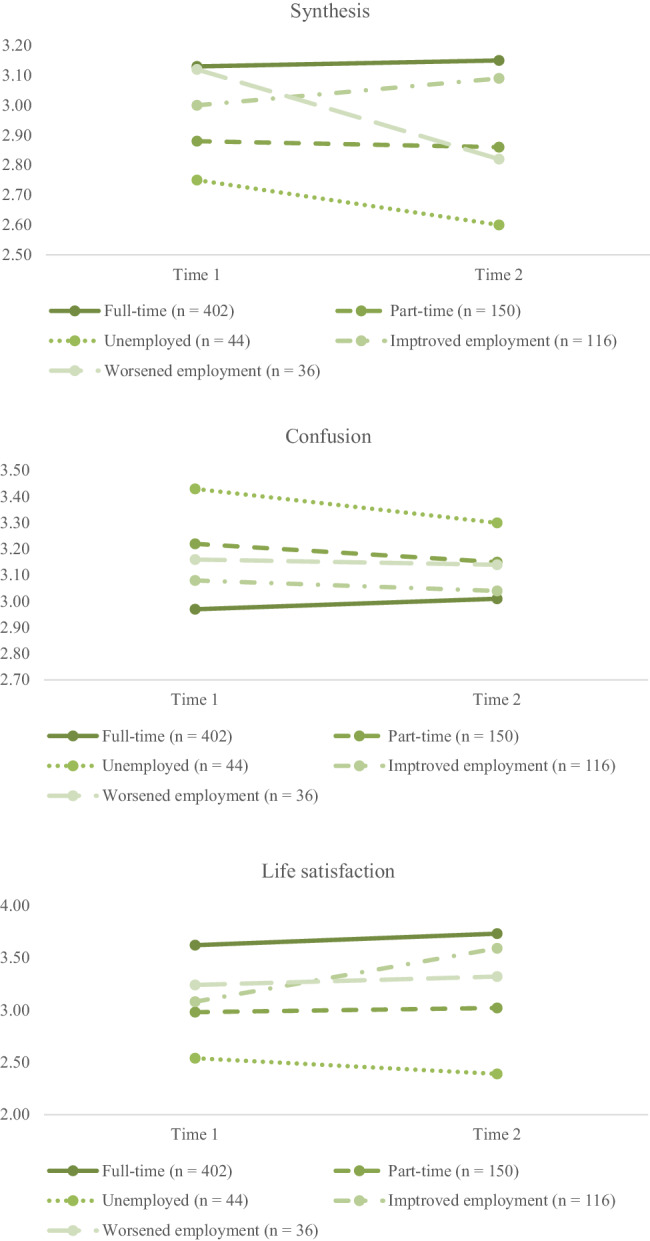
Mean Synthesis, Confusion, and Life Satisfaction Scores by Time-point and Employment Status

A two-factor 5 (employment status: full-time, part-time, unemployed, improved employment, and worsened employment) × 2 (time: T1 and T2) mixed design MANOVA was performed. The dependent variables were synthesis, confusion, and life satisfaction. Table 1 and Fig. 1 show the means and standard deviations. At the multivariate level, significant main effects of employment (Wilks’s *λ* = 0.904; *F*(12, 1960.793) = 6.350; *p* < 0.001, *ηp*^2^ = 0.033), time (Wilks’s *λ* = 0.981; *F*(3, 741) = 4.819; *p* = 0.002, *ηp*^2^ = 0.019), and time × employment status interaction (Wilks’s *λ* = 0.955; *F*(12, 1960.793) = 2.868; *p* = 0.001, *ηp*^2^ = 0.015) were found. Since statistically significant multivariate models were found, follow-up univariate ANOVA tests were conducted.

**Table 1:** Descriptive Statistics

	Full-time (*n* = 402)		Part-time (*n* = 150)		Unemployed (*n* = 44)		Improved employment (*n* = 116)		Worsened employment (*n* = 36)		Overall (*N* = 748)	
	Time 1	Time 2	Time 1	Time 2	Time 1	Time 2	Time 1	Time 2	Time 1	Time 2	Time 1	Time 2
	*M* (*SD*)	*M* (*SD*)	*M* (*SD*)	*M* (*SD*)	*M* (*SD*)	*M* (*SD*)	*M* (*SD*)	*M* (*SD*)	*M* (*SD*)	*M* (*SD*)	*M* (*SD*)	*M* (*SD*)
Synthesis	3.13 (0.74)	3.15 (0.73)	2.88 (0.73)	2.86 (0.73)	2.75 (0.88)	2.60 (1.01)	3.00 (0.73)	3.09 (0.77)	3.12 (0.72)	2.82 (0.87)	3.04 (0.75)	3.03 (0.78)
Confusion	2.97 (0.70)	3.01 (0.72)	3.22 (0.63)	3.15 (0.72)	3.43 (0.67)	3.30 (0.82)	3.08 (0.74)	3.04 (0.76)	3.16 (0.64)	3.14 (0.83)	3.07 (0.70)	3.07 (0.74)
Life satisfaction	3.62 (1.21)	3.73 (1.25)	2.98 (1.23)	3.02 (1.28)	2.54 (1.54)	2.39 (1.40)	3.08 (1.54)	3.59 (1.24)	3.24 (1.04)	3.32 (1.24)	3.33 (1.27)	3.47 (1.32)

*M* mean, *SD* standard deviation

Main effects of time were not significant for synthesis (*F*(1, 743) = 3.834; *p* = 0.051, *ηp*^2^ = 0.005) and confusion (*F*(1, 743) = 1.369; *p* = 0.242, *ηp*^2^ = 0.002). Moreover, a main effect of time was significant for life satisfaction (*F* 1, 743) = 3.907; *p* = 0.048, *ηp*^2^ = 0.005), indicating that life satisfaction score increased from T1 to T2.

There were main effects of employment status for synthesis (*F*(4, 743) = 8.234, *p* < 0.001, *ηp*^2^ = 0.042), confusion (*F*(4, 743) = 5.630, *p* < 0.001, *ηp*^2^ = 0.029), and life satisfaction (*F*(4, 743) = 19.125, *p* < 0.001, *ηp*^2^ = 0.093). Post-hoc pairwise comparisons with Bonferroni corrections were conducted. Regarding identity synthesis, individuals with full-time employment scored the highest, followed by those with part-time and improved- or worsened employments; individuals without employment scored the lowest. As for confusion, individuals with part-time and no employment scored higher than those with full-time employment. Individuals with full-time employment had the highest life satisfaction scores, followed by those with part-time and improved- or worsened employments; individuals without employment scored the lowest.

The time × employment status interaction effects were significant for synthesis (*F*(4, 743) = 2.642, *p* = 0.033, *ηp*^2^ = 0.014) and life satisfaction (*F*(4, 743) = 4.473, *p* = 0.001, *ηp*^2^ = 0.024). Regarding confusion, the interaction effect was not significant (*F*(4, 743) = 1.262, *p* = 0.283, *ηp*^2^ = 0.007; The worsened employment group showed decreased synthesis (*p* = 0.011), whereas the improved employment group showed increased life satisfaction (*p* = 0.001).

The main effect of time on identity was not significant. These results did not support Hypothesis 1, which stated that identity progressively changes in late emerging adulthood. Furthermore, the results did not support Hypothesis 2, which stated that individuals with improved employment would have increased their synthesis and decreased their confusion more than emerging adults without jobs. Moreover, the findings partially supported Hypothesis 3 that individuals with worsening employment status would have decreased synthesis or increased confusion more than emerging adults with a stable employment status. Furthermore, these results supported Hypothesis 4 that emerging adults with stable employment status would have higher synthesis and lower confusion than those without.

### Correlations Between Identity and Life Satisfaction

Table 2 shows the correlation coefficients for identity and life satisfaction at T1 and T2, along with employment status. At each time point, synthesis and confusion were positively and negatively associated with life satisfaction, respectively.

**Table 2:** Correlations Among Synthesis, Confusion, and Life Satisfaction

	1	2	3	4	5	6
1. T1 Synthesis	―	0.57^***^	−0.41^***^	−0.29^***^	0.59^***^	0.42^***^
2. T2 Synthesis		―	−0.33^***^	−0.44^***^	0.46^***^	0.62^***^
3. T1 Confusion			―	0.54^***^	−0.42^***^	−0.32^***^
4. T2 Confusion				―	−0.29^***^	−0.40^***^
5. T1 Life satisfaction					―	0.63^***^
6. T2 Life satisfaction						―

*Note*. T1, time point 1; T2, time point 2

^***^p < 0.001

### The Moderating Effects of Employment Status on the Relationship Between Identity and Life Satisfaction

To examine whether the association between identity and life satisfaction was moderated by employment status, multi-group path analysis was conducted for the five groups (employment status: full-time, part-time, unemployed, improved employment, and worsened employment; Table 3). In all groups, identity synthesis was positively associated with life satisfaction. In three groups (i.e., full-time, part-time, and worsened employment), identity confusion was negatively associated with life satisfaction. To examine the differences in path coefficients between identity and life satisfaction, a Wald test was conducted. Results showed no significant differences among all path coefficients.

**Table 3:** Relationship Between Identity and Life Satisfaction by Employment Status

	Full-time (*n* = 433)	Part-time (*n* = 162)	Unemployed (*n* = 51)	Improved employment (*n* = 123)	Worsened employment (*n* = 36)
Synthesis	0.54^***^	0.49^***^	0.62^***^	0.50^***^	0.51^***^
	[0.47, 0.61]	[0.37, 0.61]	[0.41, 0.82]	[0.35, 0.65]	[0.27, 0.76]
Confusion	−0.19^***^	−0.19^***^	−0.13	−0.10	−0.30^*^
	[−0.26, −0.11]	[−0.32, −0.06]	[−0.36, 0.10]	[−0.27, 0.07]	[−0.56, −0.04]
*R*^2^	0.41^***^	0.33^***^	0.48^***^	0.31^***^	0.52^***^

*R*^2^ = R squared; Numbers in parentheses represent 95% confidence intervals

^*^p < 0.05, ^***^p < 0.001

The findings did not support Hypothesis 5, as the relationship between identity and life satisfaction did not differ between individuals with full-time employment status and those with part-time or no employment.

## Discussion

While identity research has focused from adolescence to middle emerging adulthood, it has not been revealed how emerging adults’ identities develop in late emerging adulthood. Furthermore, it is unclear how employment status relates to identity development and the associations between identity and life satisfaction. To fill these research gaps, this study examined identity development and the associations between identity and life satisfaction in late Japanese emerging adults, focusing on the moderating effects of employment status. The study showed that identity development, life satisfaction, and employment status are intertwined during late emerging adulthood.

### Identity Development from Middle to Late Emerging Adulthood in Japan

Synthesis and confusion did not change between middle to late emerging adulthood. This finding did not support Hypothesis 1. In the *improved employment* group, there was no change in either synthesis or confusion; however, in the *worsened employment* group, synthesis decreased. These findings did not support Hypothesis 2, but partially supported Hypothesis 3. Emerging adults’ sense of self-consistency (i.e., synthesis) decreases with negative changes in employment status. One possible reason for the association between employment status and identity only in the *worsened employment* group is that the effects of getting or losing a job on self-consistency differ. In Japan, the percentage of adults changing jobs has increased since 2016 (Ministry of Health, Labour and Welfare, 2021). Even if emerging adults get a job, it takes time to determine whether the workplace is a niche for them based on their relationships with coworkers and supervisors. Contrastingly, losing a job or becoming less employable not only results in the loss of a suitable job but also raises concerns about the future with regard to money and social security. Therefore, *worsened employment* status had a greater impact on synthesis than improved ones. Regarding confusion, no association was found between the *improved*- and *worsened- employment* groups. This result suggests that confusion is constant, regardless of changes in employment status. Confusion is a stronger risk factor for psychosocial functioning than synthesis (Schwartz et al., 2017). The results confirm that confusion is a stronger and more stable trait than synthesis, which makes it less susceptible to environmental changes.

Overall, the moderating effect of employment status on identity development was not pronounced. A possible reason is that this study did not consider the period during which employment status changed. The *improved-* and *worsened-employment* groups were defined as individuals whose employment status changed in a desired or worse direction during the four-year period. Therefore, the sample may have included those who changed their job immediately after the T1 survey and those who changed closer to the T2 survey. Identity is fostered by familiarity with the work environment and the feeling that it is one’s own place (Erikson, 1968). Therefore, it can be predicted that the sense of synthesis will not be strong immediately upon entering a job but will be confirmed by further exploration within that environment. Therefore, individuals whose employment status changed near T2 may not have adapted to the workplace to the extent that they felt a sense of identity synthesis.

As expected, synthesis was higher and confusion was lower in individuals with full-time job and improved employment than for those with part-time or no job. This result supported Hypothesis 4 and suggested that individuals with a more stable employment status may have a healthier identity development. Having a full-time job is an important indicator of social approval and is crucial not only for financial security but also for the future. Specifically, in Japan, having a full- or part-time job changes the lifetime wage earned (Ministry of Health, Labour and Welfare, 2021). A high level of future security leads to a sense of temporal continuity in the self and, consequently, to a sense of synthesis. These findings suggest that employment status plays an important role in developing a healthy identity.

### The Association Between Identity and Life Satisfaction by Employment Status

The association between identity and life satisfaction was not moderated by employment status in this study. This finding did not support Hypothesis 5. It suggests that the association between identity and life satisfaction is independent of employment status. While stable employment status was expected to strengthen the association between identity synthesis and life satisfaction, attitudes toward employment status may not be uniform. Even if the emerging adults are only part-time employed or unemployed, which is considered socially undesirable, if their job or situation matches their identity, it may lead to a sense of life satisfaction. Furthermore, previous findings suggest that narrative identity (the construction of an integrated story that provides temporal and spatial coherence) predicted well-being while controlling for other variables (Adler et al., 2016). These findings suggest that the relationship between identity and life satisfaction may be highly individual and subjective.

### Developmental Implications

The current findings have two developmental implications. First, it provides evidence on identity development from middle to late emerging adulthood in the Japanese context. In a previous study, synthesis decreased and confusion increased from late-adolescence to mid emerging adulthood (Hatano et al., 2022). On the other hand, this study demonstrated variations in identity synthesis and confusion based on employment status. Emerging adults with full-time jobs showed more identity synthesis and less confusion than part-time or unemployed emerging adults. Specifically, it revealed that emerging adults who experience job loss or transition from full-time to part-time employment may encounter a decline in their sense of synthesis. These results, in conjunction with the findings of Hatano et al. (2022), indicate that identity could remain a significant developmental salient task throughout one’s twenties in Japan. Second, this study has shown that the relationship between identity and life satisfaction is independent of employment status. Regardless of employment status, individuals with high synthesis had higher levels of life satisfaction. Therefore, interventions that promote identity synthesis, regardless of employment status, may support emerging adults’ subjective well-being.

### Limitations and Future Directions

This study has several limitations. First, because identity is subjective, it was measured using self-report, which is the most important method. However, it alone cannot measure the consistency of identity with regard to behavior and attitudes. In the future, identity should be measured using other assessments and the replicability of this study’s results should be examined. Second, this study focused only on the employment status of adult carriers. While having a full-time job may be important to an adult, whether that job is the best fit for the emerging adult may be different. Future studies could further explore the relationship between work and identity by measuring, in addition to employment status, whether emerging adults are making successful career choices and examining identity development from the combination of the two. Third, this study focuses only on employment status in examining the moderating effect between identity and life satisfaction. There may be other factors that affect the relationship between identity and life satisfaction, such as sex and individual job satisfaction. In the future, it is expected that the relationship between employment status, identity, and life satisfaction will be better understood by considering the combination of these factors in addition to employment status. Fourth, the direction of association between identity and life satisfaction was not examined. As cross-lagged effect models are increasingly criticized (Lucas, 2023), it is necessary to examine the direction of associations using a random intercept cross-lagged panel model, which examines the direction of associations at the within-person level using longitudinal data at three or more time points (Hamaker et al., 2015). In the future, additional survey time points should be included, and identity development and life satisfaction should be examined in relation to intra-individual changes. Fifth, the survey was limited to emerging adults from Japan. While being “Japanese” is a strength because identity develops in context, the differences in identity development in emerging adults from other countries are unclear. In the future, it will be necessary to compare the aspects of identity development in emerging adults in other countries.

## Conclusions

Identity develops during late emerging adulthood and is associated with life satisfaction. Employment status moderates this development. To disentangle these complex developmental relationships, this study examined identity development and its association with life satisfaction, focusing on the moderating effects of employment status. The results indicated that emerging adults who lost their jobs or had worsening employment status had decreased synthesis. Furthermore, individuals with a more stable employment status were more synthesized and less confused than those without. Additionally, regardless of employment status, higher synthesis was associated with higher life satisfaction. Overall, these findings provide evidence on the employment status-dependent heterogeneity of identity development in late emerging adulthood as well as the employment status-independent homogeneity of the association between identity and life satisfaction. Furthermore, this study provides evidence that contributes to theoretical and empirical research on the development of identities in emerging adults.

### Supplementary Information

Supplementary_Figure_Tables

## Data Availability

This manuscript’s data will not be submitted. The datasets generated and analyzed during the current study are not publicly available but are available from the corresponding author upon reasonable request.
