# Supplementary material for: Does Employment Status Matter for Emerging Adult Identity Development and Life Satisfaction? A Two-wave Longitudinal Study
Source: J Youth Adolesc. 2024 May 15;53(9):2097–107. doi: 10.1007/s10964-024-01992-x (PMC11333549; doi:10.1007/s10964-024-01992-x)
Supplement: Supplementary file 1 — Supplementary_Figure_Tables [file 10964_2024_1992_MOESM1_ESM.docx]

Title:- Does Employment Status Matter for Emerging Adult Identity Development and Life Satisfaction? A Two-wave Longitudinal Study

Journal name:- *Journal of Youth and Adolescence*

**Figure S1**

*Measurement Models for Identity and Life Satisfaction*

*
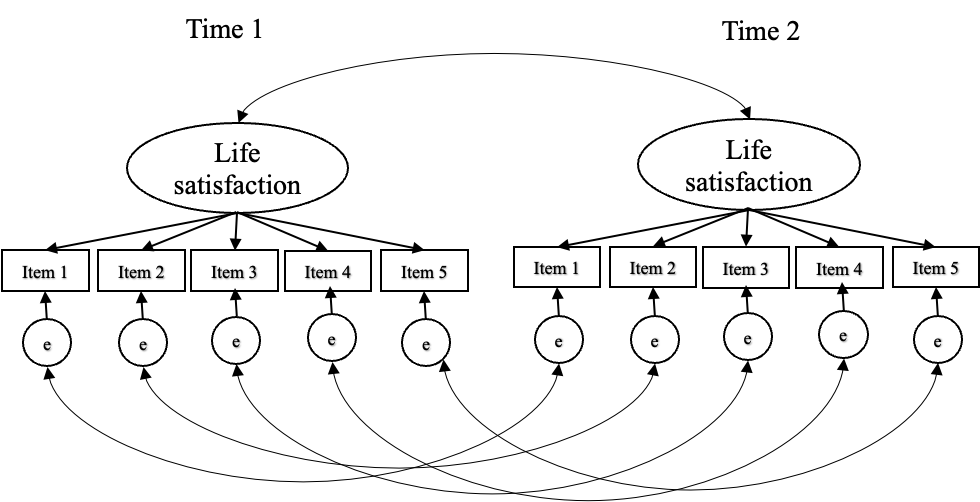
*

**Table S1**

*Participants' Region, Household Income, and Educational Background*

|  | % |
| --- | --- |
| Region |  |
| Urban |  |
| Kanto | 33.5 |
| Chubu | 19.9 |
| Kansai | 16.9 |
| Less urban |  |
| Hokkaido | 4.9 |
| Tohoku | 6.4 |
| Chugoku | 5.5 |
| Shikoku | 2.1 |
| Kyusyuu | 10.9 |
| Household income |  |
| Low (<2 million yen) | 9.0 |
| Middle (2 to 8 million yen) | 45.5 |
| High (>8 million yen) | 10.1 |
| Unknown | 18.4 |
| Educational background |  |
| Master's | 8.2 |
| Bachelor's | 55 |
| Associate degree | 6.1 |
| Diploma | 10.7 |
| High school | 17.6 |
| Junior high school | 2.3 |
| Other | 0.1 |

**Table S2**

*Fit Indices of the EPSI and Life Satisfaction Measurement Model*

|  | S-Bχ^２^ | CFI | RMSEA [90% CI] | Compared to configural model | | | | |
| --- | --- | --- | --- | --- | --- | --- | --- | --- |
|  |  |  |  | ΔS-Bχ^２^ | *Δdf* | *p* | \|ΔCFI\| | \|ΔRMSEA\| |
| EPSI |  |  |  |  |  |  |  |  |
| Configural invariance | 250.486 | .951 | .045 [.039-.052] |  |  |  |  |  |
| Metric invariance | 258.138 | .950 | .044 [.038-.051] | 6.678 | 6 | .352 | .001 | .001 |
| Full scalar invariance | 270.865 | .949 | .043 [.036-.049] | 16.484 | 15 | .351 | .002 | .002 |
| Life satisfaction |  |  |  |  |  |  |  |  |
| Configural invariance | 70.417 | .988 | .040 [.028-.053] |  |  |  |  |  |
| Metric invariance | 77.286 | .987 | .039 [.028-.051] | 5.350 | 4 | .253 | .001 | .001 |
| Full scalar invariance | 94.065 | .984 | .041 [.031-.052] | 24.480 | 9 | .004 | .004 | .001 |

*Note*: S-Bχ^２^ = Satorra–Bentler adjusted χ^２^test statistic; ΔS-Bχ^２^ = change in S-Bχ^２^; *p* = *p* value across model comparisons, based on S-Bχ^２^ difference; testing (ΔS-Bχ^２^); CFI = comparative fit index; RMSEA = root mean-square error of approximation; 90% CI = 90% confidence interval; Δ= change in parameter; EPSI = the Erikson Psychosocial Stage Inventory
